# Facilitating a smoother transition to renewable energy with AI

**DOI:** 10.1016/j.patter.2022.100528

**Published:** 2022-06-10

**Authors:** Joyjit Chatterjee, Nina Dethlefs

**Affiliations:** 1 Department of Computer Science and Technology, University of Hull, Cottingham Road, Hull HU6 7RX, UK

## Abstract

Artificial intelligence (AI) can help facilitate wider adoption of renewable energy globally. We organized a social event for the AI and renewables community to discuss these aspects at the International Conference on Learning Representations (ICLR), a leading AI conference. This opinion reflects on the key messages and provides a call for action on leveraging AI for transition toward net zero.

## Main text

Global energy-related carbon emissions soared to an alarming level in 2021, re-bounding to the second-highest rate of annual rise in history.^1^ To prevent the recurrence of this dire phenomenon, it is integral to position our future energy ecosystem around renewable energy. Currently, the share of electricity in global final energy consumption is around 20%, predicted to rise to a whopping 50% by 2050 (e.g., through transition to electric vehicles). Wind and solar energy can meet the majority of global energy demands and entirely displace fossil fuels by 2050.^2^

While renewable energy sources like wind energy can facilitate the rapid transition to a low-carbon economy, there are several hurdles that hinder the wider adoption of such energy sources at a global scale.^3^ Some barriers result from a socioeconomic divide, such as offshore wind energy presently being very Europe-centric, with comparatively minimal developments in the Global South (where high-initial costs of setting up offshore wind farms are challenging to sustain) despite enormous potential for wind power, e.g., in India and Brazil. Other roadblocks arise on ethical and moral grounds, such as public concerns about the visual effect of wind turbines on the environmental landscape, threat to wildlife during accidental collisions with turbine blades, and greenhouse gases emitted during manufacturing of solar panels. However, the biggest hurdle from an operational perspective is the frequent operational inconsistencies and failures in complex engineering systems like wind turbines and solar panels. They lead to intermittent power production and thus affect the availability and reliability of such energy systems for powering cities and communities.

Most modern renewable energy systems collect operational data from their electrical and mechanical subsystems that are equipped with multiple sensors. Such data include metrics such as power production and site performance in solar panels, measured external environmental conditions, and health status of internal subcomponents in wind turbines and so forth. Such data present several opportunities for the data science and artificial intelligence (AI) community; for instance:

• Images of solar panels (or turbine blades) can be used for applying computer vision techniques to facilitate AI-driven inspection and analysis of defects in the components.
• Historical failure data from sensors in wind turbines (or solar panels) can be used to develop AI models for predictive and corrective maintenance, helping engineers quickly fix (or avert) potential failures in the systems and their subcomponents.
• Historical power production data (e.g., in solar panels and wind farms) can be used to develop AI models to assist energy system operators on better planning for unit commitments and integration with the electrical grid, thus preventing unexpected outages.

The examples mentioned above are not exhaustive: these are some of the major challenges that AI can help tackle in the renewables domain. Despite the variability in where and how AI can be leveraged, the common factor in applying AI to the renewables domain is decision support. By leveraging historical data optimally, AI can provide a robust accumulation of historical domain-specific trends into an intelligent model. This can help aid engineers and technicians in the routine inspections, maintenance activities, planning ahead for outages, and so forth, often much in advance of such problems becoming unmanageable. For instance, an unexpected failure in a critical wind turbine subcomponent like the gearbox, or a fault in an inverter in a solar energy system, can lead to severe economic losses for the operators and significant downtime and repair/replacement costs. AI can help predict such incipient failures and also suggest maintenance actions to fix and/or avert faults, thus bringing down operations and maintenance (O&M) costs and reducing downtimes as well as increasing the lifetime of components in renewable energy systems. Ultimately, AI in the age of Industry 4.0 can help renewable energy operators provide customers (including households and industries) with affordable and reliable electricity from complex energy systems while also accelerating the transition to sustainability.^4^

### We are witnessing negligible impact of AI in the renewables domain

Despite the plethora of opportunities discussed above, there is negligible focus in applying AI in the renewables domain, both in the industry (currently, most energy system operators rely on signal processing or vibration analysis techniques during O&M for condition monitoring) as well as academia (fewer than 100 papers have been published in applying AI for O&M of renewables in the last 5 years).^5^ In academia, both conventional machine learning (ML) models (e.g., support vector machines [SVMs], decision trees) as well as deep learners (e.g., transformers, long short-term memory networks [LSTMs]) have been developed for tasks ranging from fault prediction in internal subcomponents of renewable energy systems to long-term power forecasting,^6^ with deep learners often outperforming conventional models in recent years in terms of their predictive accuracy. However, there is a significant gap between the development versus the actual adoption of such models in the real world by the industry.

The industry is reluctant to use AI in practice as it cannot get a sophisticated understanding of risks when utilizing such models, especially high-performing (but more complex) black-box models like deep learners on the face of uncertain outputs. Higher-quality research in academia is limited by the lack of openly available data to train models in the renewables domain, particularly information such as O&M manuals, sensor measurements, and historical power production forecasts, which are generally commercially sensitive to the industry. Additionally, such data widely vary in the renewables domain at present, with a lack of consistency and standard practices (e.g., naming conventions of alarms during faults in solar panels vary across energy operators, and recorded parameters in wind turbines from the same operator located at different sites can be inconsistent), which poses significant challenges in pre-processing such information for actually training AI models. While such challenges could potentially be tackled by generating synthetic simulated data to train the AI models, such data fail to provide a transparent picture on the unexpectedly changing real-world scenarios (e.g., sudden faults and downtimes) in renewable energy systems. This leads to a vicious cycle, with the industry being reluctant to share data and academics unfortunately being left behind in developing AI models that can be calibrated and adapted to real-world data. With this closed loop, there is a lack of the balance that is needed to nurture well-thought-out research that can deliver immediate value to the industry.

Given the fast-paced nature of AI, many state-of-the-art models that are released are not explored well in the short term, and potential pitfalls and a deeper understanding of such models is only evident in the long term. This also practically prohibits the industry from leveraging high-performing off-the-shelf models in the short term for real-time decision support, and they are restricted to continue utilizing more conventional techniques based on signal processing and vibration analysis for O&M. While leveraging explainable AI models instead of black-box learners can help develop better trust and confidence in the industry, such models either originate from a very different domain (e.g., healthcare) or are slightly less performing compared with black-box models, thus making them inconsistent in producing exactly similar results in a new domain like renewables.

Leveraging high-performing deep learners generally requires significant computational resources, and, indirectly, carbon emissions are generated during training. This also creates a trade-off in using AI in the renewables domain, wherein there is a need to strike a balance when transitioning to a low-carbon future. This also requires more robust governmental policies as well as both natural and moral support from the public, which is currently not consistently present in this promising domain.

### Call for action

As in any other discipline, AI is not a “silver bullet” for the renewables domain, and it comes with its own opportunities and risks when leveraged toward transition to net zero. In itself, AI cannot deliver any viable change unless we put in place robust policies and practices supported willingly by all members of the community. This includes governments that are willing to create sustainability policies and citizens (including industry, academia, and the general public) who are well informed and educated on where and how AI can make a difference in the low-carbon transition process.

A better understanding of how the industry can best collaborate with academia would significantly help to accelerate the uptake of AI and ensure that models transition from academic laboratories to being put into practice in the industry during O&M. This in turn would help reduce the gap between those who develop state-of-the-art AI models (academia) and those who actually use these models in the renewables domain (industry), while smoothening the difficulties and strengthening the interaction between them. This process needs to arise in the form of a global movement that has to be systematic with the sharing of resources and technology to ensure that low- and middle-income nations are not left behind in this race. Additionally, there are social problems that need to be addressed to realize a greater impact, which can potentially be done through education and knowledge exchange with the public, driven by the AI and the renewables community, particularly pertaining to the benefits of renewables versus their negative consequences. Based on interactions with academic and industry experts as well as community-gathered insights, the social webpage^7^ provides a comprehensive list of various open-source resources in the renewables domain that can be used to train AI models for decision support during O&M.

It is integral for AI to be regarded as a broader umbrella in the renewables domain, beyond the lens of neural networks, which are currently the trend in the AI community.^8^ Some of the potentially valuable areas of focus, both in terms of the models and in terms of the broader family of ML processes, are:

(1) For the renewables community, optimization is extremely important (e.g., during O&M planning and dispatch of vessels to carry out repairs in offshore wind farms, planning the layout of solar panels for generating optimal power). Reinforcement learning has vast potential in facilitating better decision making during O&M, and the AI community needs to work together with the renewables community in different ways to support it.
(2) The majority of applications of AI in the renewables domain currently pertain to power forecasting (regression task) and fault prediction at a binary or multi-class level (classification task). A greater focus on natural language generation can significantly help to develop better trust and confidence in predictive AI models, providing engineers and technicians with detailed descriptions and analyses of faults as well as suggesting domain-specific suitable fixes for the faults in the form of human-intelligible O&M messages. Such techniques can be better supported by utilization of multimodal data sources such as domain-specific knowledge graphs curated by domain experts for explainable decision support during O&M.
(3) To tackle the challenges posed by limited data availability for developing AI models (either due to their commercially sensitive nature or in situations when newer renewable energy systems have not been in operation for long), transfer learning techniques can be leveraged to extrapolate the learnt knowledge in other energy systems to newer scenarios. This includes extrapolating such knowledge across wind turbines located at different sites (e.g., offshore and onshore) and solar panels of different technical specifications. Additionally, there are also avenues for few-shot or zero-shot learning to curate the formulation of high-performance learners in the face of few or no data.
(4) It can be extremely beneficial for the AI community to integrate the traditional ML workflow with statistically robust physics-based models to develop better trust and confidence in the learning process. This can also help make the safety-critical application of O&M more robust to uncertain outcomes and predictions that are made by traditional AI models on the face of new, unseen data (e.g., during rarer fault types and operational inconsistencies in energy systems as a result of forced outages or requested shutdowns).

It is integral for energy system operators to transition to a data-driven approach and strive for a long-term goal to achieve an AI-driven transformation during O&M in the future. This calls for energy companies and original equipment manufacturers (OEMs) to invest greater time and resources into AI as well as data curation, storage, and dissemination practices (including focusing on development of industry-wide uniform data strategies and policies). It would also help realize the optimal potential of the benefits AI has to offer to the renewables industry. AI would not replace human engineers and technicians, and thus not eliminate existing jobs or create redundancy through automation, but would help support them in their everyday decision-making process during O&M by making it more intelligent and efficient; this can be accomplished by AI delivering novel insights and helping domain experts look into the future based on learnt trends in historical data. Optimal value of AI during the energy transition can only be realized in a collaborative environment between ML models and human domain experts during decision support. In summary, it is integral for the AI community to work together with the renewables community to realize this vision; this can include community-building social events, workshops, stronger interactions between academia and industry, and interplay of governments and intergovernmental organizations like the United Nations, in both digitization as well as energy sectors.

Note that this transition should follow the principles of frugality, allowing the development of AI solutions in a resource-constrained manner that does not compromise the end goal of facilitating a sustainable future. This includes taking into account the environmental effects of training large-scale AI models and potentially widening of the digital divide between developed and developing countries. To realize a frugal data-driven approach, it is vital for the AI community to focus on the problem first rather than the solution and only utilize more complex models (such as deep learners) when simpler ML models do not work. We envisage that following such a well-planned yet flexible approach in utilizing AI in the renewables domain will help facilitate a smooth and efficient transition to a low-carbon economy in the next few decades.
